# c-Abl Inhibitors in Parkinson’s: Exploring Hypotheses on Alpha-Synuclein Modulation

**DOI:** 10.34172/apb.42806

**Published:** 2025-02-09

**Authors:** Jyutia Nargish, Hirok Jyoti Baishya, Piyong Sola

**Affiliations:** Department of Pharmacology, NETES Institute of Pharmaceutical Science, NEMCARE Group of Institutions, Shantipur, Mirza, Kamrup, Assam, India.

**Keywords:** Parkinson’s disease, Neurodegeneration, c-Abl, α-synuclein, Phosphorylation

## Abstract

Parkinson’s disease (PD) stands as the second most prevalent neurodegenerative disorder, impacting a global population estimated between 6 to 10 million individuals. The condition primarily arises from a dopamine deficiency and the presence of α-synuclein, forming Lewy bodies in the substantia nigra pars compacta (SNcp). Despite the ongoing quest to unravel the precise pathophysiological mechanisms underlying PD, recent literature reviews posit that heightened activation of the Abelson non-receptor tyrosine kinase(c-Abl), in brain tissues plays a pivotal role in neurodegeneration observed in PD patients. Notably, these studies put forth compelling evidence suggesting that c-Abl inhibitors’ interventions exhibit notable therapeutic potential. The potential benefits encompass enhancements in motor function, prevention of dopamine neuron loss, and the meticulous regulation of α-synuclein phosphorylation and clearance. These findings collectively advocate for the exploration of c-Abl as a prospective therapeutic target, thereby presenting inhibitors of this kinase as promising candidates for intervention in the management of PD.

## Introduction

 Parkinson disease (PD) is a chronic, progressive condition that affects 1% of people over 60.^1^ Primarily PD characterized by the early and pronounced degeneration of dopaminergic cells in the substantia nigra pars compacta (SNpc), PD manifests as a neurological disorder, leading to a deficit of dopamine in the basal ganglia and resulting in a movement disorder akin to parkinsonism.^2^ The process of neurodegeneration in PD is concomitant with the occurrence of cytoplasmic inclusions, known as Lewy bodies (LBs), and neuritic inclusions termed Lewy neurites (LNs). These inclusions manifest in surviving dopaminergic neurons and other affected regions within the central nervous system (CNS).^3^ The precise mechanisms underlying the degeneration of dopaminergic neurons in the SNpc in PD remain elusive. Multiple factors, including protein misfolding and aggregation, compromised protein clearance pathways, cell-autonomous processes, and the phenomenon of “prion-like protein infection,” are implicated in the pathogenesis of PD. Additionally, potential contributors encompass mitochondrial damage, energy insufficiency, oxidative stress, and excitotoxicity, thereby emphasizing the multifaceted nature of processes that may contribute to the development of this neurological disorder.^4^

 The manifestations of this condition can be categorized into two primary domains: motor features and non-motor symptoms. Motor features encompass a spectrum of clinical presentations, notably characterized by bradykinesia, gait disturbance, tremor, rigidity, and speech deficits. On the other hand, non-motor symptoms encompass a diverse array of manifestations, including but not limited to depression, hyposmia, cognitive impairment, sleep disorders, and constipation. This comprehensive classification facilitates a nuanced understanding of the multifaceted nature of the observed clinical presentations in individuals affected by the condition.^5^

###  c- Abl and tyrosine kinase

 Kinases have emerged as a promising class of drug targets for PD therapy. Studies have highlighted the significant role of disrupted kinase activity and altered phosphorylation pathways in the development and progression of PD.^6^ The only other member of the Abl family of non-receptor tyrosine kinases is Arg (ABL2; Abl-related gene), which includes c-Abl (ABL1; Abelson tyrosine kinase). The c-Abl protein, with a molecular weight of approximately 120 kDa, is a member of the cytoplasmic tyrosine kinase family.^7^ Structurally c-Abl consist of N-terminal Src-homology domain 2 (SH2) and Src-homology domain (SH3).^8^ The c-Abl protein exhibits a high degree of evolutionary conservation across metazoan organisms and is characterized by ubiquitous expression within various subcellular compartments, including the cytoplasm, nucleus, mitochondria, and endoplasmic reticulum.^7^ The c-Abl protein demonstrates a broad spectrum of functional interactions, engaging with an array of cellular components such as cell signaling adaptors, kinases, and phosphatases.^7,9^ It has been demonstrated recently that SNCA induced neurodegeneration is mostly caused by dysregulated activation of c-Abl.^10^ Numerous stimuli, such as growth hormones, chemokines, oxidative stress, DNA damage, adhesion receptors, and microbial pathogens, closely control endogenous Abl kinases.^11,12^ Upon activation, Abl kinases control signaling pathways involved in cytoskeletal remodeling, which play a crucial role in cellular protrusion formation, cell migration, morphogenesis, adhesion, endocytosis, and phagocytosis.^13^ Through a number of pathways, such as the activation of parkin, the aggregation of α-synuclein (SNCA), the activation of microglia, and the activation of protein kinase C delta, the c-Abl tyrosine kinase contributes significantly to the pathophysiology of PD. As a result, the neuroprotective potential of c-Abl inhibitors in treating PD has been investigated in a number of preclinical investigations.^14^ Table 1 presents an overview of the procedures and results from these investigations.

**Table 1:** Overview of the researches that looked into how c-Abl inhibition might protect animal models against PD

**Animals**	**Intervention**	**Conclusion**	**References**
Adult mice of the wild type with conditional neural knockout of c-Abl. Age-matched mice of the wild type.	At two-hour intervals, mice were given four intraperitoneal injections of either saline or MPTP (20 mg/kg). Seven days later, the c-Abl activity was measured use an antibody against p-tyrosine 245 c-Abl. Additionally, the levels of FBP1 and AIMP2 were measured by using certain antibodies to target them.	One crucial posttranslational alteration that inhibits parkin's activity is its phosphorylation by c-Abl, which may be a factor in the causes of sporadic PD. Furthermore, PD may be treated by inhibiting c-Abl, which may have a neuroprotective effect.	Ko et al, 2010^15^
Adult male wild type mice	Alpha-synuclein or lentiviral c-Abl were stereotaxically injected into the substantia nigra of mice on both sides. Following three weeks of lentiviral injection c-Abl, half of the rats received dimethyl sulfoxide alone for an additional three weeks, while the other half received a daily dose of 10 mg/kg of nilotinib dissolved in dimethyl sulfoxide. Following animal sacrifice, brain tissues were prepared for neurochemical analysis.	Alpha-synuclein particles are autophagically cleared by nilotinib in several brain regions, including the striatum, hippocampus, and the brain.	Hebron et al, 2013^16^
Male C57Bl/6 mice aged 7-8 weeks.	At two-hour intervals, mice were given four intraperitoneal injections of either saline or MPTP. They received either a vehicle or a nilotinib injection three days later. Mice were then tested for motor coordination and behavior. They subsequently got an intraperitoneal a lethal dosage of pentobarbital, and samples of their brains were prepared for neurochemical analysis.	Nilotinib increases the effectiveness of dopaminergic signalling by preventing cyclin-dependent kinase 5 (Cdk5) from becoming phosphorylated. Additionally, it works in concert with D1 agonists to boost the expression of the c-fos gene. When administered systemically, it normalizes aberrant motor activities brought on by MPTP.	Tanabe et al, 2014^17^
Adult mice of the wild type with conditional neural knockout of c-Abl	Mice were given four intraperitoneal injections at one-day intervals of 30 mg/kg imatinib, 5 mg/kg SB203580 (a particular inhibitor of the p38-mitogen activated protein kinase (MAPK) pathway), or saline (12 hours prior to and following the MPTP injection). After that, mice received four intraperitoneal injections of MPTP at a dose of 20 mg/kg. The animals were killed seven days following the final MPTP injection, and the striatum was ready for western blot examination.	Cell death caused by oxidative stress is mediated by C-Abl and its substrate p38α. Imatinib-induced c-Abl knockout reduced MPTP-induced dopaminergic loss of neurons.	Wu et al, 2015^18^

###  Hypotheses

 c-Abl protein is involved in various biological processes, including the coordination of cellular growth and survival, modulation of integrin signaling, facilitation of actin polymerization, and the regulation of cell migration. From several literature reviews we found that c-Abl level is adjusted in postmortem striatum and autophosphorylation of c-Abl at y412 enhanced the catalytic activity in the substantia nigra and striatum of the PD patient. Phosphorylation of c-Abl protein increases in the SNCA overexpression and result in SNCA aggregation. Therefore, the inhibition of the c-Abl protein may prevent SNCA accumulation and can be useful in the management of PD.

###  Justification for the hypotheses

**Figure 1:**
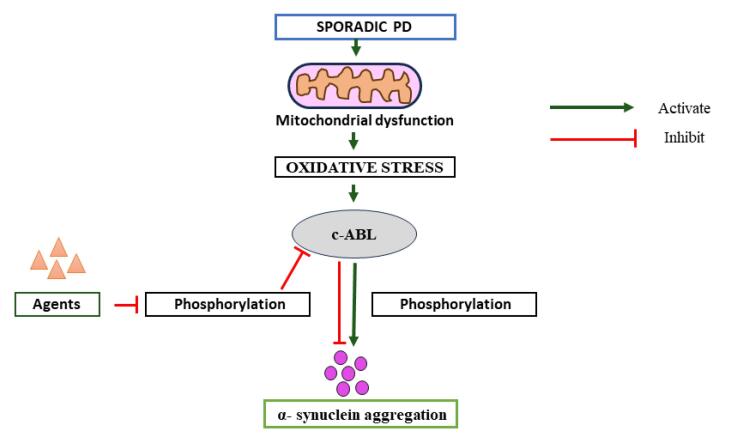
Pathway of Parkinson’s disease; significance of c-ABL inhibitor on progression of disease

 Pathologically aggregated conformations of SNCA constitute a prominent causative factor in the degeneration of dopamine neurons within the substantia nigra, contributing significantly to the observed pathological changes.^19-21^ Elevated c-Abl activity, quantified by the ratio of phosphorylated Y245 c-Abl to total c-Abl, is discernible in the postmortem brains of individuals afflicted with PD, particularly within the substantia nigra and striatum. In the human brain, SNCA is typically phosphorylated at Ser129 only to a limited extent under normal conditions.^22,23^ A significant buildup of pS129 SNCA is observed in transgenic animal models and the brains of individuals with PD. These results suggest that pS129 may represent the pathological or toxic form of the protein.^22,24,25^ The discovery that c-Abl mediates alternate phosphorylation sites on SNCA, namely at Tyr39 (pY39) and Tyr125 (pY125), raises the possibility that c-Abl is involved in the development of neurodegenerative disorders.^10,26^ These brain regions are known to be pathologically implicated in PD.^10,15,27^ The activation of c-Abl, as gauged by phosphorylation at the (Ser^129^) pS129 site, is likewise evident in postmortem brains of individuals diagnosed with PD.^16,28-30^ These observations suggest a discernible correlation between the activation of c-Abl and the pathological manifestations associated with PD.^10^ The kinase c-Abl operates within a rigorously regulated framework, with activation occurring in response to either oxidative or genotoxic stress. Moreover, the functional efficacy of this protein is contingent upon its precise subcellular localization.^31-33^ In a recent study by Hebron et al, compelling evidence was presented elucidating a bidirectional interplay between SNCA and c-Abl in vivo. Research indicates that c-Abl activation may be a critical factor in driving neurodegeneration, as c-Abl knockout or chemical inhibition has shown neuroprotective effects in animal models of PD. The findings delineate a reciprocal relationship, wherein elevated SNCA expression prompts the phosphorylation and subsequent activation of c-Abl. Conversely, an augmentation in c-Abl expression and activity leads to the accumulation of SNCA. These observations suggest that the inhibition of c-Abl may represent a promising therapeutic strategy for mitigating SNCA accumulation and safeguarding against SNCA-induced toxicity in the context of PD^34^ (Figure 1).

## Conclusion

 c-Abl, a central orchestrator of critical cellular functions, undergoes meticulous regulation to maintain its basal activity. Interestingly, the aberrant activation of c-Abl is observed in postmortem brain tissue from PD patients, specifically within regions marked by pathological neurodegeneration. c-Abl activation, triggered by reactive oxygen species, links to cell death in PD, revealing its dual role in neurodegeneration and oxidative stress regulation. Evidence suggests c-Abl activation, not just SNCA accumulation, initiates neurodegeneration. Our hypothesis proposes intracellular pathogenic SNCA transformation via c-Abl activation triggers neurodegenerative cascade. Inhibiting c-Abl activation emerges as a potential disease-modifying strategy for PD.

## Competing Interests

 All the authors declare that they have no known competing financial interests or personal relationships that could have appeared to influence the work reported in this paper. The authors declare that they have no conflict of interest.

## Consent for Publication

 Not applicable.

## Ethical Approval

 Not applicable.
